# Ectopic parathyroid adenoma in the soft palate: a case report

**DOI:** 10.1186/s40463-016-0165-z

**Published:** 2016-10-18

**Authors:** Brent A. Chang, Anil Sharma, Donald W. Anderson

**Affiliations:** 1 Division of Otolaryngology–Head & Neck Surgery, University of British Columbia, 2775 Laurel St., 4th floor Otolaryngology (ENT), Vancouver, BC V5Z 1M9 Canada; 2 Division of Otolaryngology–Head & Neck Surgery, University of Saskatchewan, Saskatoon, Canada

**Keywords:** Ectopic, Parathyroid adenoma, Case report, Hyperparathyroidism

## Abstract

**Background:**

Ectopic parathyroid adenomas can occur in numerous anatomic locations. While ectopic parathyroid adenomas can rarely occur in the pharyngeal region, this has not previously been described in the soft palate.

**Case presentation:**

We report the first case of ectopic parathyroid adenoma within the soft palate. A 59 year old woman presented with hyperparathyroidism. She remained persistently hyperparathyroid after initial parathyroidectomy. Repeat exploration for a lesion suspicious on PET-CT for an ectopic parathyroid adenoma in the parapharyngeal region was unsuccessful in treating the hyperparathyroidism. An ectopic adenoma in the soft palate was eventually discovered. Removal through a transoral approach was successful in treating the hyperparathyroidism.

**Conclusions:**

Ectopic parathyroid adenomas can occur in various anatomical locations that may be missed even with the use of the various imaging modalities. The soft palate should be added to the list of possible ectopic locations high in the neck.

## Background

The existence of ectopic parathyroid tissue remains a significant hurdle in the surgical management of hyperparathyroidism. Embryological development and migratory descent to the lower neck predisposes the parathyroid glands to ectopic locations. Ectopic adenomas have been reported to account for anywhere from 4 to 16 % of patients with hyperparathyroidism and are thought to be the cause of a significant portion of failed primary surgery for hyperparathyroidism [1–3].

Ectopic parathyroid glands have been classically described as occurring in numerous anatomic locations anywhere from the angle of the mandible to the mediastinum [4–6]. Most commonly they occur in the mediastinum, in the path of the vagus nerve and recurrent laryngeal nerve, and within the thyroid parenchyma [7]. Several reports have described ectopic parathyroid adenomas in more unusual locations, such as within the hypoglossal nerve [6], posterior triangle of the neck [8, 9], axilla [10] and pericardium [11]. Lesions can less commonly occur higher in the pharyngeal structures with only two reports describing lesions in either the oropharynx or nasopharynx [1, 12]. We present a case of a diagnostically challenging ectopic parathyroid adenoma in the soft palate, which has not previously been described.

## Case presentation

A 59 year old woman with a 7 year history of recurrent nephrolithiasis and mild hypertension presented with a complaint of weakness and fatigue with associated bone and joint pain. Further history revealed that she had had five previous episodes of urinary calculi requiring surgery or lithotripsy. She was also discovered to have had a first degree atrioventricular block and was currently awaiting pacemaker insertion. There was no family history of thyroid/parathyroid disorders or history of previous radiation. Physical examination was unremarkable. Laboratory investigations revealed hypercalcemia (2.89 mmol/L), hypophosphatemia (0.78 mmol/L) and elevations in parathyroid hormone (PTH) (15.2 pmol/L). Bone mineral density studies revealed cortical bone osteoporosis.

A ^99m^Tc-sestamibi scan did not reveal any abnormality. An exploratory parathyroidectomy was then arranged and the right and left superior parathyroid glands were removed, along with a localized biopsy of the left inferior parathyroid gland. The right upper gland was 1 cm in diameter and was thought to be an adenoma. No inferior right parathyroid gland was identifiable. She subsequently remained hyperparathyroid and underwent re-exploration and removal of the previously biopsied left inferior gland. Again, a right inferior gland was not identified and a right thyroid lobectomy to locate a possible intra-thyroid adenoma was performed. The final pathological analysis of the removed thyroid lobe did not identify any parathyroid tissue within the thyroid parenchyma, but there was normal parathyroid tissue in the paratracheal fat removed at the time. Parathyroid hormone levels continued to remain persistently elevated. Although subsequent MRI and CT scans remained negative, PET-CT scanning showed a 3 cm region of uptake in the right parapharyngeal region behind the thyroid cartilage at the level of the arytenoids. Selective venous sampling showed increased PTH levels in this area.

A third parathyroid exploration was undertaken for elective resection of the suspicious area shown on PET-CT. Frozen section pathology results suggested the presence of inflammatory tissue secondary to a suture granuloma from the previous neck exploration. Intraoperative PTH levels continued to remain elevated. An exhaustive search of the neck was then undertaken, including skeletonization of the carotid artery to the level of the bifurcation, mobilization of the jugular vein, identification of the course of the vagus nerve through the neck, dissection down to the cervical fascia and prevertebral fascia and mobilization of the pharynx and esophagus from the prevertebral fascia. At this point, it was thought that the lesion might be in the upper neck. A secondary incision in the upper neck was considered, but the decision was made to defer further exploration.

**Fig. 1:**
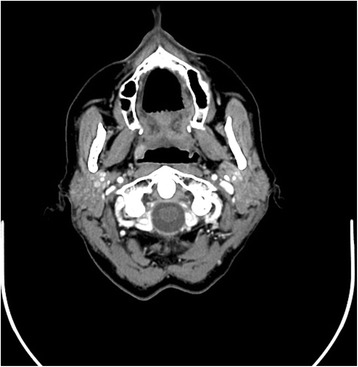
This image shows a contrast CT scan of the neck, axial view, demonstrating a suspicious mass in the lateral right soft palate

Post-operatively, the patient continued to have elevated serum calcium and PTH and repeat venous sampling study was performed, again confirming positive uptake in the proximal right internal jugular vein. A repeat CT scan of the head and neck (Fig. 1) was obtained; which revealed a 10 × 5 mm mildly enhancing submucosal soft tissue lesion within the soft palate extending into the tonsillar pillar suggestive of a parathyroid adenoma.

The patient underwent a successful transoral surgical removal of the lesion. Pathology confirmed the presence of a parathyroid adenoma. As expected, the patient became hypocalcemic postoperatively.

## Discussion

The occurrence of ectopic parathyroid adenomas can typically be explained by their embryological development [7, 12–14]. The parathyroid glands develop from the third and fourth pharyngeal pouches. The superior parathyroid glands develop from the fourth pharyngeal pouch, where as the inferior parathyroid glands arise from the third pharyngeal pouch and descend a further distance with the thymus, which migrates into the mediastinum. Ectopic parathyroid glands can thus occur anywhere along the embryologic descent of the parathyroid glands. Specifically, the parathyroid glands originate from epithelial cells at dorsal and ventral wings at the distal extremities of the third and fourth pharyngeal pouches. During normal development, the parathyroid glands must separate and subsequently travel in a caudal direction [15]. It can be postulated that if this separation did not occur early on, that a parathyroid gland could end up high in the pharyngeal wall. However, because the inferior parathyroid glands are assisted in their migration by being pulled caudally by the thymus, such a developmental error might be rare. The lack of capsular fixation of the parathyroid glands also makes them susceptible to ectopy and unusual anatomic migrations [5].

We are aware of only a few reports describing ectopic parathyroid adenomas in very superior locations. In a series of 288 patients with persistent hyperparathyroidism, Jaskowiak et al. described one patient with a lesion in the wall of the nasopharynx near the nasal septum and one patient with a lesion high in the vagus nerve at the level of the C1-C2 vertebrae [1]. Chan et al. reported on a small series of patients with ectopic adenomas in the pharyngeal or surrounding structures [12]. A cervical approach was used for all of these lesions, except for one case in which a mandibular osteotomy was used.

There are sparse reports in the literature of utilizing a transoral approach to resect ectopic parathyroid adenomas. The standard surgical approach for re-exploration following persistent hyperparathyroidism is transcervical [1, 16]. A high oblique neck incision is also used when an undescended parathyroid adenoma is suspected [1, 16]. Transoral surgical approaches have been briefly described for non-ectopic parathyroid surgery, but are still in the experimental stages [17, 18]. Transoral robotic surgery approaches have also been described for certain ectopic parathyroid adenomas [19].

Reoperation for persistent hyperparathyroidism can be challenging. This case demonstrates some of the challenges of surgical management of persistent hyperparathyroidism. Normal anatomy and tissue planes are often disrupted and sometimes difficult to recognize. While ectopic parathyroid adenomas are thought to be the most common cause of persistent hyperparathyroidism following neck exploration, a number of reasons other than ectopic tumors can be responsible for persistent hyperparathyroidism, such as disease recurrence, surgical inexperience, missed or residual tissue, parathyromatosis and supernumerary glands. Given these challenges, localization studies are recommended prior to reoperation [1, 2].

Previous reports in the literature describe an algorithm for the management of persistent hyperparathyroidism [19, 20]. Similarly, at our institution, the general approach to persistent hyperparathyroidism after previous surgery involves a careful review of preoperative imaging, operative notes, and pathology followed by ultrasound and a ^99m^Tc-sestamibi scan. If localization remains unsuccessful, cross-sectional imaging is employed (e.g. CT or MRI). Selective venous sampling for parathyroid hormone levels is done only after previous unsuccessful parathyroid exploration. ^11^C-methionine PET scanning has been very useful in localizing parathyroid adenomas that have failed localization by other techniques (data not yet published). Intraoperative parathyroid hormone monitoring and frozen section are useful at the time of re-exploration.

The case illustrates several points regarding diagnostic imaging for hyperparathyroidism. Most commonly, ^99m^Tc-sestamibi scan and ultrasound are used as first-line imaging modalities [21]. CT and MRI are generally used as a second line imaging choice. In this patient, ^99m^Tc-sestamibi scan, CT, MRI, PET-CT, and PTH selective venous sampling were all utilized. PET-CT, ^99m^Tc-sestamibi scan, MRI and even the initial CT scan failed to localize this ectopic adenoma. Invasive techniques such as selective venous sampling are typically reserved for cases where non-invasive imaging has failed to yield an answer; however, this case highlights the potential utility of such methods. Details regarding the specific utility and pitfalls of these various imaging modalities are described elsewhere [22].

## Conclusions

This case demonstrates some of the challenges of surgical management of persistent hyperparathyroidism. In diagnostically challenging cases of persistent hyperparathyroidism, unusual ectopic locations of parathyroid adenomas must be considered. Such consideration is necessary in order to avoid numerous re-operative explorations and the associated risks to the patient. Instead of the upper range of parathyroid glands being stated as the angle of the mandible, we suggest that the parathyroid adenomas should be considered possible at least as high as the nasopharynx. Given the high ectopic potential of these glands, the upper limiting position may not yet be known.

## Abbrevations

CT: Computed Tomography
MRI: Magnetic Resonance Imaging
PET-CT: Positron Emission Tomography–Computed Tomography
PTH: Parathyroid hormone
